# Molecular Screening for Potential Resistance to Toxic Invasive Prey: Implications for Island Conservation

**DOI:** 10.3390/genes17101286

**Published:** 2026-10-15

**Authors:** Alisa Tobe, Masako Izawa, Miho Inoue-Murayama

**Affiliations:** 1 Wildlife Research Center, Kyoto University, Kyoto 606-8203, Japan; t.alisa210@gmail.com; 2 Faculty of Science, University of the Ryukyus, Okinawa 903-0213, Japan; izawa@cs.u-ryukyu.ac.jp

**Keywords:** cane toad, cardiotonic steroids, target-site insensitivity, molecular evolution, conservation genetics, island conservation, convergent evolution

## Abstract

**Background/Objectives:** Invasive species can disrupt native predator–prey interactions, and toxic invasive prey may pose particular threats to native predators. The cane toad (*Rhinella marina*) secretes cardiotonic steroids (CTSs) that can cause lethal poisoning in predators. We reviewed the molecular basis and evolutionary patterns of CTS resistance and evaluated the potential and limitations of genetic screening for assessing wildlife vulnerability on Ishigaki-jima and Iriomote-jima Islands, Japan. **Methods:** We reviewed experimental, structural, and evolutionary evidence for CTS resistance associated with Na+/K+-ATPase α subunit (NKAα) and screened publicly available NKAα1 sequences from vertebrates occurring on the two islands, focusing on amino acid states previously associated with CTS target-site insensitivity. **Results:** NKAα1 sequences were available for 96 of 185 native terrestrial vertebrate species. Most retained the putatively ancestral Q111/N122 combination, whereas alternative states at these major resistance-associated sites occurred in only a few species. These included T111/N122 in the black-faced spoonbill (*Platalea minor*), and E111/N122 in the crested serpent-eagle (*Spilornis cheela perplexus*) and grey-faced buzzard (*Butastur indicus*). T111 has shown context-dependent effects on CTS sensitivity in functional assays, whereas E111 has been proposed as a candidate resistance-associated state but lacks direct functional validation. The endangered Iriomote cat (*Prionailurus bengalensis iriomotensis*), an endemic predator that frequently consumes anurans, retained Q111/N122. **Conclusions:** NKAα1 sequence alone cannot establish whole-animal susceptibility or conservation risk because resistance may depend on sequence background, other NKAα paralogs, physiological mechanisms, and behavior. Molecular screening should therefore be viewed as an initial tool for identifying candidate molecular signatures and prioritizing species and knowledge gaps for subsequent functional, toxicological, and ecological investigation.

## 1. Introduction

The negative impacts of invasive species on native ecosystems have long been recognized. Invasive species can affect native animals through multiple mechanisms, including predation, competition, habitat alteration, disease transmission, and incorporation into native food webs as novel prey [1,2,3,4,5,6]. Island ecosystems are considered particularly vulnerable to biological invasions because their geographical isolation is often associated with small populations, restricted distributions, and limited opportunities for recolonization following population declines [7,8]. Understanding how invasive species affect island ecosystems is therefore crucial for developing effective conservation strategies.

However, vulnerability to invasive species can vary substantially among native species because responses to novel organisms depend partly on pre-existing behavioral, ecological, and physiological traits shaped by their evolutionary history [9,10]. Identifying such species-specific vulnerability is therefore important for predicting which native species are most likely to be affected by biological invasions. When invasive species are toxic, differences in physiological susceptibility among native species may strongly influence their vulnerability to invasion [11,12].

The cane toad (*Rhinella marina*) is listed among the World’s 100 Worst Invasive Alien Species and has been widely introduced across tropical and subtropical regions worldwide, particularly on islands in the Caribbean, Pacific, and Asia-Pacific regions, as well as in Australia [13,14]. Cane toads possess parotoid glands that secrete bufadienolides (BDs), a group of cardiotonic steroids (CTSs) [15]. These toxins can cause lethal poisoning in native predators that consume cane toads, as extensively documented in Australia [16]. Cane toads have also been introduced to several island regions of Japan, including Chichijima and Hahajima in the Ogasawara Islands, Minami-Daitojima, and Ishigaki-jima Island [17,18,19,20]. Ishigaki-jima Island is located in the southernmost part of the Ryukyu Archipelago, Japan, where cane toads became established following their initial introduction in 1977 [21]. On Ishigaki-jima Island, the native frog-eating snake (*Lycodon rufozonatus walli*) has been reported to die while swallowing cane toads [22]. Despite longstanding concerns regarding the toxic effects of cane toads on native predators [17], the susceptibility of the broader native predator community on Ishigaki-jima Island remains largely unknown.

**Figure 1:**
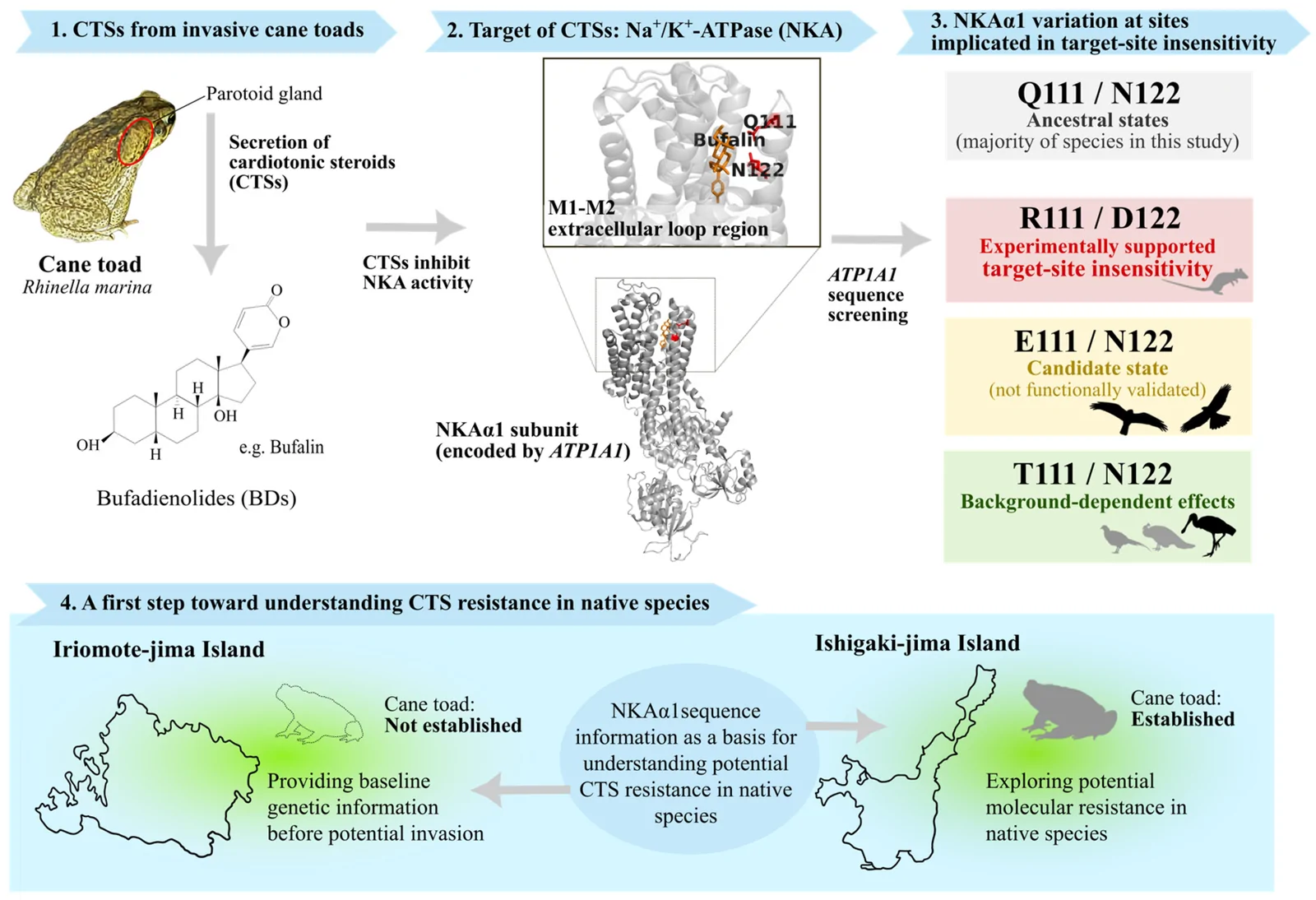
Conceptual framework illustrating the screening of Na+/K+-ATPase α1 subunit (NKAα1) amino acid sequences as a first step toward understanding cardiotonic steroid (CTS) resistance in terrestrial vertebrates. Cane toads (*Rhinella marina*) secrete bufadienolides (BDs), a group of CTSs that inhibit NKA. Bufalin is shown as a representative BD. The NKA structure is based on Sus scrofa (PDB ID: 4RES), with the M1–M2 extracellular region enlarged to highlight amino acid positions 111 and 122, which have repeatedly been implicated in naturally evolved target-site insensitivity. The NKAα1 subunit is encoded by *ATP1A1*, the gene examined in this study. Among the NKAα1 sequences examined, most species retained the ancestral Q111/N122 combination. R111/D122 was identified in the black rat (*Rattus rattus*), and E111/N122 in the crested serpent-eagle (*Spilornis cheela*) and grey-faced buzzard (*Butastur indicus*). T111/N122 was detected in the black-faced spoonbill (*Platalea minor*), Indian peafowl (*Pavo cristatus*), and common pheasant (*Phasianus colchicus*). These amino acid combinations differ in the strength of evidence supporting their association with CTS resistance. The lower panel illustrates the contrasting invasion status of cane toads on the two islands. Cane toads are established on Ishigaki-jima Island but have not become established on Iriomote-jima Island. Molecular screening provides preliminary genetic information relevant to understanding potential CTS resistance in native vertebrates in these different ecological contexts. Gray silhouettes represent invasive species, whereas the other silhouettes represent native species. Animal silhouettes were obtained from PhyloPic (https://www.phylopic.org/ (accessed on 14 September 2026)).

In contrast, cane toads have not become established on adjacent Iriomote-jima Island, located only approximately 20 km from Ishigaki-jima Island (Figure 1). Nevertheless, the island remains at risk of future invasion, and a total of 11 individual cane toads have been detected and removed since 2000 [17,23]. Ishigaki-jima and Iriomote-jima Islands are relatively large and mountainous islands within the Ryukyu Archipelago, with areas of approximately 222 and 289 km^2^ and maximum elevations of 526 and 470 m, respectively. Both are characterized by a warm, humid subtropical climate with high annual precipitation (1991–2020; Japan Meteorological Agency). Their relatively large areas and topographic complexity support diverse habitats, which, together with the unique biogeographic history of the Ryukyu Archipelago, contribute to high levels of species diversity and endemism [24,25]. Iriomote-jima Island, in particular, forms part of a UNESCO Natural World Heritage site and supports numerous endemic and threatened species. Assessing the vulnerability of its native fauna to toxic invasive cane toads before their establishment is therefore important for proactive conservation.

One potential first step toward assessing such vulnerability is to examine genetic variation associated with physiological responses to toxins. Resistance to CTSs has evolved convergently across diverse animal lineages through amino acid substitutions in the α subunits of Na+/K+-ATPase (NKAα). These subunits are encoded by *ATP1A* genes and constitute the primary molecular targets of CTSs [26,27,28,29,30]. Previous studies have demonstrated that substitutions at positions Q111 and/or N122 in the first extracellular loop between transmembrane helices M1 and M2 of NKAα, which contributes to the CTS-binding site, play important roles in reducing CTS sensitivity. Although amino acid substitutions in NKAα can provide molecular evidence of potential target-site insensitivity, whole-animal responses to CTSs depend on multiple additional factors, including pharmacokinetic and pharmacodynamic processes [31]. Genetic screening should therefore be viewed as an initial means of identifying candidate species for further investigation rather than as a direct predictor of physiological susceptibility. Indeed, the crested serpent-eagle (*Spilornis cheela perplexus*), an endangered endemic resident subspecies of Ishigaki-jima and Iriomote-jima Islands [32,33], is known to consume cane toads and possesses a Q111E substitution in NKAα1 [34,35]. However, the potential susceptibility or resistance of most other vertebrate predators occurring on these islands remains unknown.

We first summarize the history of research into the molecular basis of NKAα-associated CTS resistance. We then compile publicly available NKAα1 sequence information for resident terrestrial vertebrates occurring on Ishigaki-jima and Iriomote-jima Islands and screen for amino acid states previously associated with CTS resistance. Finally, we evaluate the strengths and limitations of using such molecular information as an initial step in conservation vulnerability assessment, with particular emphasis on proactive conservation of Iriomote-jima Island before the establishment of invasive cane toads (Figure 1).

## 2. History of the Identification of Residues Associated with Toxin Resistance

### 2.1. History of the Experimental Studies

Experimental studies have identified numerous amino acid residues in NKAα subunits that can alter sensitivity to CTSs, particularly ouabain. Early site-directed mutagenesis studies demonstrated that replacing residues Q111 and N122, located near the extracellular end of the M1–M2 region, markedly reduced ouabain sensitivity (Table 1; [30,36]). Subsequent site-directed and random-mutagenesis studies identified additional sensitivity-associated residues distributed across several transmembrane and extracellular regions, demonstrating that the molecular determinants of CTS sensitivity extend well beyond sites 111 and 122 (Table 1; [37,38,39,40,41,42]).

Structural studies subsequently provided a mechanistic basis for this broad distribution of experimentally identified sensitivity-associated residues. Earlier biochemical studies established a ten-transmembrane-segment topology of the NKAα [43], and the first crystal structure of NKA established the overall arrangement of these ten transmembrane helices [44]. Subsequent structural analyses of ouabain-bound NKA showed that ouabain occupies a cavity extending from the extracellular surface into the transmembrane domain [45,46,47]. In the high-affinity complex, this binding pocket is formed primarily by M1, M2, M4, M5, and M6, together with residues near its extracellular entrance [46]. These structural observations are broadly consistent with mutagenesis studies showing that alterations at spatially distributed residues can influence ouabain binding and sensitivity (Table 1). Yang et al. further summarized at least 41 sites in NKAα implicated in CTS sensitivity based on site-directed mutagenesis and structural studies [48].

### 2.2. Naturally Occurring Substitutions

In contrast to the broad range of residues that can experimentally alter CTS sensitivity, naturally evolved target-site insensitivity repeatedly involves a much narrower subset of NKAα residues. Comparative studies across multiple insect orders have revealed striking parallelism in cardenolide-adapted insects, with substitutions strongly concentrated in the first M1–M2 extracellular loop, particularly at positions 111 and 122, despite the much larger number of sites potentially capable of modulating cardenolide sensitivity (Table 2; [48,49,50,51,52,53]). Functional assays further demonstrated that substitutions at positions 111 and 122 can act synergistically to increase ouabain resistance [49,54]. Within milkweed butterflies, sequence comparisons and enzyme assays demonstrated a stepwise reduction in cardenolide sensitivity associated with successive changes at positions 111 and 122 during the evolution of Danaini [51]. Evolutionary solutions are not completely restricted to point substitutions at these positions; for example, resistant *ATP1A* copies in pyrgomorphid grasshoppers contain both substitutions and a two-amino-acid insertion in the first extracellular loop, and functional assays demonstrated that the derived loop sequence confers strong cardenolide insensitivity [55]. A survey across six insect orders detected evidence of positive selection at site 111 in cardenolide-sequestering lineages with a single *ATP1A* copy and at sites 115, 118, and 122 in lineages possessing neofunctionalized *ATP1A* duplicates [48].

Convergence within the M1–M2 region has also been reported in vertebrates, although the NKAα paralog involved can differ among taxa [27,28,29,56,57]. In particular, Ujvari et al. examined the α3 subunit in squamate reptiles and found that resistant toad-feeding lineages repeatedly evolved L111 and R120, with the same combination arising independently on at least four occasions [29]. Their broader comparison of insect α subunits, amphibian and mammalian α1 subunits, and reptilian α3 subunits highlighted recurrent resistance-associated changes at position 111, together with positions 119, 120, and 122 [29].

The repeated use of these residues does not, however, imply that their phenotypic effects are independent of the surrounding sequence background. Comparative analyses of CTS-adapted insects identified sites 111 and 122 as recurrent targets associated with specialization on CTS-producing plants, while variation at site 119 was strongly associated with substitutions at site 111 [52,53]. Reconstruction of their evolutionary histories, combined with genome editing of the endogenous *ATP1A* locus in *Drosophila melanogaster*, further demonstrated that epistatic interactions among these sites can shape mutational trajectories by ameliorating antagonistic pleiotropic effects [53]. Evidence that resistance-conferring substitutions must be considered in the context of other amino acid changes also exists in vertebrate NKAα1. In Leptodactylus frogs, one copy of a duplicated *ATP1A1* evolved resistance to toad toxins, while the other retained ancestral susceptibility [27]. Twelve amino acid differences were maintained between the resistant and susceptible paralogs despite recurrent gene conversion, and protein-engineering experiments demonstrated that two substitutions at the major resistance-associated sites 111 and 122 substantially increased toxin resistance, whereas the remaining ten substitutions collectively mitigated deleterious effects on ATPase activity [27]. These findings demonstrate that the evolution of a functional resistant NKAα can involve both substitutions that directly increase target-site insensitivity and additional substitutions that compensate for their functional costs. Together, these findings indicate that the recurrent involvement of sites such as 111 and 122 in CTS adaptation cannot be interpreted independently of the surrounding sequence background, because their evolutionary and functional effects may be shaped by pleiotropic and epistatic interactions with other residues [27,48,53].

These considerations place important limits on inferring CTS resistance from amino acid sequence alone. Positions 111 and 122 have particularly strong functional and comparative support as determinants of CTS sensitivity and therefore provide useful primary indicators of potential target-site insensitivity [29,30,49]. However, their phenotypic effects can depend on the broader sequence background, and resistance therefore cannot be inferred from these positions in isolation [27,52,53].

### 2.3. Investigating Potential CTS Resistance Using Molecular Information

Accordingly, in this review, we treat amino acid states in the M1–M2 extracellular loop, particularly positions 111 and 122 of NKAα1, as primary indicators of potential CTS target-site insensitivity in vertebrates of Ishigaki-jima and Iriomote-jima Islands, while also examining other residues for which experimental evidence indicates effects on CTS sensitivity or interactions with resistance-conferring substitutions. Comparative evidence of repeated evolution in CTS-adapted lineages is used as additional support when evaluating these candidate sites (Table 2; [48,49,50,53]). We therefore distinguish substitutions with direct functional evidence for altered CTS sensitivity from candidate modifying or compensatory substitutions, rather than categorically assigning species as resistant or susceptible solely on the basis of positions 111 and 122. This framework permits systematic screening for amino acid states previously associated with target-site insensitivity, while allowing cautious consideration of sequence backgrounds that may modify the phenotypic effects or evolutionary accessibility of resistance-associated substitutions. Because such epistatic effects are strongly dependent on genetic background and cannot be established from comparative sequence data alone, substitutions without direct functional validation are treated as candidates and are not themselves interpreted as evidence of CTS resistance.

## 3. Identification and Comparative Analysis of NKAα1 in Native Vertebrates on Iriomote-jima and Ishigaki-jima Islands

### 3.1. Methods

#### 3.1.1. Dataset

We considered 199 terrestrial vertebrate species occurring on Iriomote-jima and Ishigaki-jima Islands for molecular screening. These comprised six native mammals, 17 native reptiles, eight native amphibians, 154 native birds, one invasive mammal, six invasive reptiles, two invasive amphibians, three invasive birds, and two captive mammals (Table S1; [58,59,60,61,62]). The native mammals, reptiles, and amphibians included all species known to occur on the two islands, whereas the native birds were restricted to resident breeders, migrant breeders, and winter visitors [58]. The vertebrate faunas of the two islands are largely similar, although Iriomote cat and several bird species occur on only one of the two islands (Table S1).

#### 3.1.2. Molecular Screening

In this review, we focused exclusively on *ATP1A1* because it is ubiquitously expressed in most vertebrate tissues [63], and its coding sequences could be recovered relatively consistently across the taxa examined. *ATP1A2* and *ATP1A3* were not included in the comparative screen because complete, confidently assigned sequences could not be obtained consistently across the sampled taxa.

For each species, we first searched the National Center for Biotechnology (NCBI) for publicly available *ATP1A1* nucleotide sequences. Searches were conducted at the species level, irrespective of the geographic origin of the sequenced individual; thus, the sequences used were not necessarily derived from individuals collected on Iriomote-jima or Ishigaki-jima Island. When an annotated *ATP1A1* nucleotide sequence was unavailable, we recovered the *ATP1A1* coding sequence from the corresponding genome assembly using Miniprot v0.18, which performs protein-to-genome alignment [64]. When multiple genome assemblies were available for a species, higher-level and more contiguous assemblies were preferentially selected based on assembly level and N50 statistics.

The human (*Homo sapiens*; NP_000692.2) and chicken (*Gallus gallus*; NP_990852.2) NKAα1 protein sequences were used as queries for mammalian and avian genomes, respectively, because NKAα1 is well characterized in these model species. For reptilian genomes, for which no comparable model reference was available, the NKAα1 protein sequence from a phylogenetically closely related species (*Sceloporus undulatus*; XP_042305353.1) was used as the query. Miniprot was run with the --gff-only option and otherwise default parameters. The resulting gene models were exported in GFF format, and coding sequences were extracted from the corresponding genome assemblies using GffRead v0.12.7.

When multiple candidate *ATP1A1* sequences were recovered from the same genome assembly, they were compared at both the nucleotide and amino acid levels. If multiple nucleotide sequences encoded identical amino acid sequences, a single representative sequence was retained for subsequent analyses. Candidate sequences were translated into amino acid sequences and aligned using MAFFT v7.505 with the L-INS-i algorithm. Residue positions were numbered according to the human NKAα1 sequence. Sequences containing ambiguous amino acid states, apparent gene-prediction errors, or less than 90% alignment coverage were excluded.

To confirm that the retained candidate sequences represented NKAα1 rather than other NKAα paralogs, we inferred a maximum-likelihood phylogeny using IQ-TREE v3.1.3 [65]. The best-fitting amino acid substitution model was selected using ModelFinder, and branch support was assessed using 1000 ultrafast bootstrap replicates. The phylogenetic analysis included the candidate protein sequences together with human NKAα2, NKAα3, and NKAα4 (NP_000693.1, NP_689509.1, and NP_653300.2, respectively) and chicken NKAα2 and NKAα3 (NP_990807.2 and NP_990806.1, respectively) as reference paralogs. Only candidate sequences that clustered within the reference NKAα1 clade, rather than with NKAα2 or NKAα*3*, were retained for subsequent analyses.

To screen for potential molecular signatures of CTS target-site insensitivity, we first examined amino acid states at positions 111 and 122, which have the strongest experimental and comparative support for recurrent involvement in CTS resistance (Section 2). We then extended the screening to the 41 NKAα residues compiled by Yang et al. (2019) as sites potentially affecting cardenolide sensitivity (Table 1; [48]). Because the inclusion of a residue among these 41 sites does not imply that every amino acid substitution at that position has been functionally demonstrated to confer CTS resistance, we separately compared the amino acid states observed in our dataset with specific substitutions that have been experimentally tested for effects on CTS sensitivity or NKA function (Table 2). Experimentally tested substitutions were distinguished from amino acid states for which evidence was limited to comparative or sequence-based observations. In accordance with the framework described in Section 2, substitutions lacking direct functional validation were treated as candidate molecular signatures and were not themselves interpreted as evidence of CTS resistance.

### 3.2. Results

#### 3.2.1. NKAα1 Sequence Availability and Validation

**Figure 2:**
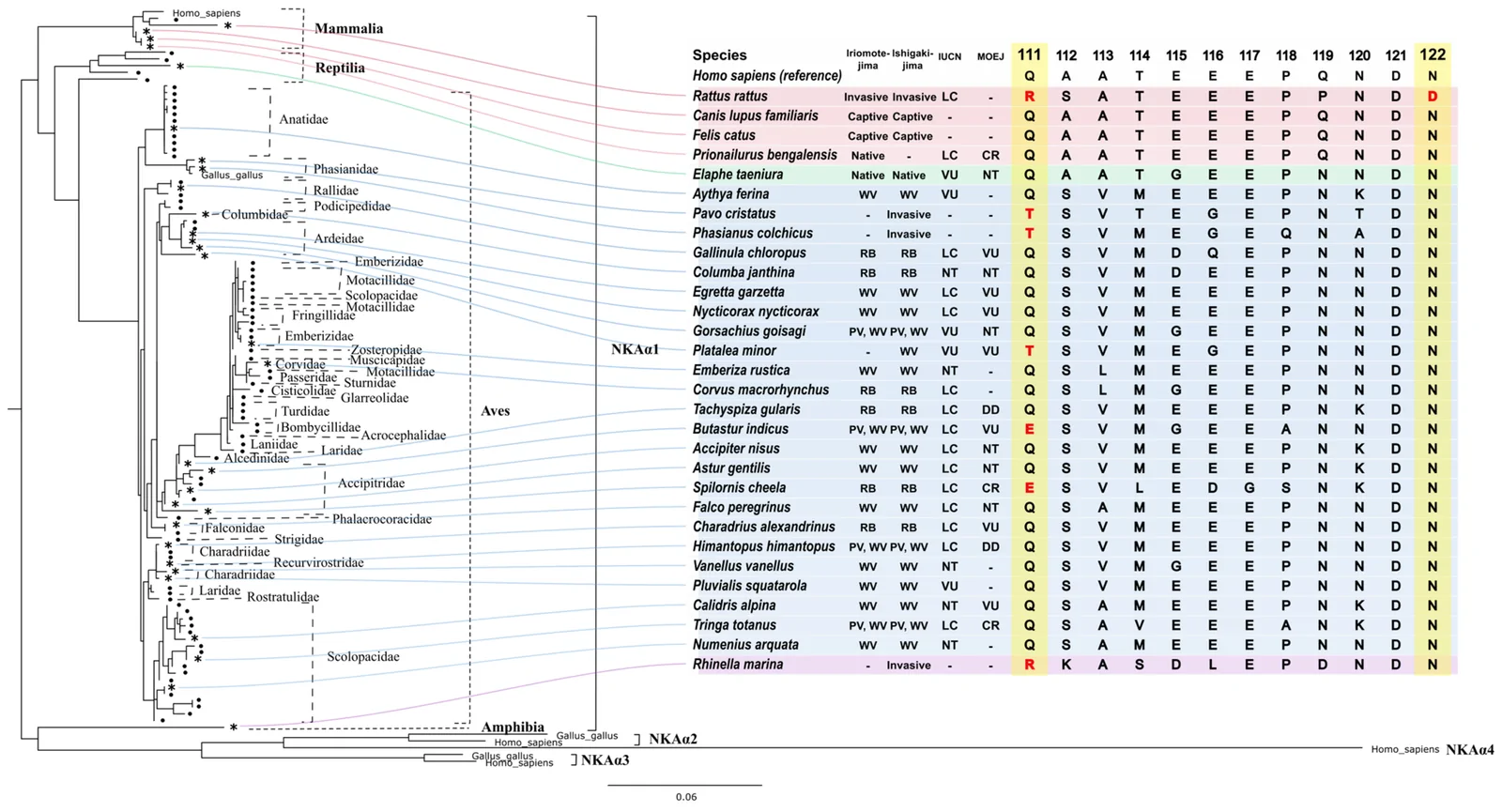
Phylogenetic distribution of NKAα1 amino acid variation at positions 111–122 among the vertebrates examined in this study. The maximum-likelihood phylogeny includes NKAα1 sequences from the examined vertebrates and reference NKAα paralogs used to assess orthology. Amino acid residues at positions corresponding to residues 111–122 of human NKAα1 are shown for selected species in the accompanying annotation table. Positions 111 and 122, which have repeatedly been implicated in the evolution of CTS target-site insensitivity, are highlighted in yellow. Amino acid states differing from the human NKAα1 sequence at these positions are shown in red. Occurrence status on Iriomote-jima and Ishigaki-jima Islands and conservation status according to the IUCN Red List and the Ministry of the Environment, Government of Japan (MOEJ), are shown for each species in the annotation table. The background colors of the annotation table indicate vertebrate classes (mammals, reptiles, birds, and amphibians). Solid lines distinguish NKAα paralogs (NKAα1, NKAα2, NKAα3, and NKAα4), while dashed lines with narrow and wide gaps distinguish taxonomic classes and orders, respectively. Asterisks (*) indicate species included in the annotation table, whereas dots (•) indicate species included in the phylogeny but not displayed in the annotation table. Colored lines connect the asterisk-marked species in the phylogeny to their corresponding rows in the annotation table, with line colors matching the vertebrate classes. Branch lengths represent estimated amino acid substitutions per site.

NKAα1 sequences were available for 96 of the 185 native species included in the survey (51.9%), with sequence availability varying among taxonomic groups: 92 of 154 birds (59.7%), two of six mammals (33.3%), two of 17 reptiles (11.8%), and none of the eight amphibians (Table S1). Alignment coverage, calculated as the proportion of non-gap positions relative to the total alignment length, ranged from 92.4% to 98.4% (mean, 97.9%) among the retained NKAα1 sequences (Supplementary File S1). Phylogenetic analysis including reference NKAα paralogs from human and chicken confirmed that the identified sequences clustered with NKAα1 rather than with the other NKAα paralogs (Figure 2; Supplementary File S2).

#### 3.2.2. Amino Acid Variation at Sites Potentially Relevant to CTS Sensitivity

Most vertebrate NKAα1 sequences retained the putatively ancestral Q111/N122 combination, including 22 of the 23 endangered species examined (Figure 2; Table S1; Supplementary File S1). However, several alternative amino acid states were observed at position 111. Two invasive galliform birds, the common pheasant (*Phasianus colchicus*) and Indian peafowl (*Pavo cristatus*), and the black-faced spoonbill (*Platalea minor*; IUCN: VU; MOEJ: VU), a winter visitor to Ishigaki-jima Island, possessed T111 while retaining N122. The crested serpent-eagle possessed E111/N122, consistent with our previous report [34]. The same E111/N122 combination was also identified in the grey-faced buzzard (*Butastur indicus*). Among mammals, the invasive black rat (*Rattus rattus*) possessed the R111/D122 combination, for which direct experimental evidence of reduced CTS sensitivity is available [30]. This species has also been reported to prey on cane toads in Australia [66]. Overall, variation at these two major resistance-associated sites was concentrated primarily at position 111, whereas position 122 was highly conserved among the vertebrates examined.

We next extended the comparison to the 41 NKAα1 residues compiled by Yang et al. [48] as sites potentially affecting cardenolide sensitivity based on mutagenesis, structural analyses, and related functional studies. Despite their potential functional relevance to cardenolide sensitivity, amino acid variation at many of these sites showed no clear association with taxa previously suggested to possess CTS resistance (Tables S2 and S3; [48]). Some amino acid states corresponding to experimentally investigated derived states were widespread among the vertebrates examined. For example, M114 and S874 were present in 88 and 96 species, respectively. Conversely, many other experimentally implicated positions were highly conserved across the vertebrate NKAα1 sequences. The widespread occurrence of some experimentally investigated amino acid states indicates that their presence alone cannot be interpreted as evidence of naturally evolved CTS resistance, although potential effects on CTS sensitivity cannot be excluded.

Finally, several amino acid states corresponding to substitutions examined in functional studies summarized in Table 2 were detected in the vertebrate NKAα1 sequences examined here (Tables S4 and S5). Among species with an alternative amino acid state at position 111, the crested serpent-eagle, grey-faced buzzard, and cane toad also carried amino acid states corresponding to substitutions included in the broader sequence background experimentally examined in *Leptodactylus* NKAα1 [27] (Tables S4 and S5).

### 3.3. Discussion

#### 3.3.1. A Restricted Set of Molecular Signatures Associated with Potential CTS Resistance Among Island Vertebrates

Our comparative analysis supports the distinction between the broad biochemical potential for altering CTS sensitivity and the much narrower set of molecular changes repeatedly associated with naturally evolved target-site insensitivity [48]. The ancestral Q111/N122 combination predominated among the vertebrates examined, whereas alternative amino acid states at these positions occurred in only a limited number of lineages. Given the strong functional and comparative evidence for positions 111 and 122 [48,49,51,55], these sites provide useful first-line indicators of potential NKAα1-mediated target-site insensitivity, although amino acid states at these positions should not be treated as definitive predictors of organismal resistance.

The different amino acid states observed at positions 111 and 122 also warrant different levels of confidence in their interpretation. R111/D122 has strong experimental support as an ouabain-insensitive NKAα1 state [30], whereas T111, observed in the two invasive galliform birds (common pheasant and Indian peafowl) and the black-faced spoonbill, has been reported repeatedly in cardenolide-adapted insects. Experimental studies, however, indicate that its effect on CTS sensitivity is strongly dependent on the surrounding amino acid background [49]. Q111T alone produced little or no detectable increase in ouabain resistance in a Drosophila NKAα background, whereas its combination with N122H conferred substantially greater resistance [49,54]. To our knowledge, none of these three species has been reported to prey on cane toads on Ishigaki-jima Island. Further functional and ecological investigation is therefore required to determine whether T111 contributes to CTS target-site insensitivity in these species and whether it has any relevance to their responses to cane toad toxins.

Q111E, observed in the crested serpent-eagle and grey-faced buzzard, has also been reported in NKAα1 of the black-headed grosbeak (*Pheucticus melanocephalus*), a specialized predator of monarch butterflies that sequester cardenolides from milkweed [26]. Groen and Whiteman proposed that this substitution may contribute to target-site insensitivity, noting that Q111E was absent from 157 other passerine species examined that do not consume milkweed [26]. The documented consumption of cane toads by the crested serpent-eagle provides additional ecological context for a possible association between E111 and CTS target-site insensitivity. However, direct functional evidence for an effect of Q111E is currently lacking, and its phenotypic effect may depend on the surrounding sequence background [52,53]. In this context, E116D and I279V in the crested serpent-eagle and I135V in the grey-faced buzzard are of particular interest. The cane toad also carried L180Q and I279V, and all of these amino acid states correspond to substitutions included in the broader sequence background experimentally examined in *Leptodactylus* NKAα1 [27]. In *Leptodactylus*, these background substitutions collectively mitigated the functional costs associated with resistance-conferring substitutions [27]. Although their individual functional effects remain unknown, the occurrence of these amino acid states in both the predatory species examined here and the cane toad further highlights the need to consider the broader sequence background when evaluating candidate resistance-associated substitutions.

#### 3.3.2. Evolutionary Context of Q111E in Avian Species

The identification of Q111E in the grey-faced buzzard expands the known distribution of this amino acid state among raptors. In our previous comparison of nine raptor species, Q111E was detected only in the crested serpent-eagle, whereas the other species, including its close relative the black-chested snake-eagle (*Circaetus pectoralis*), retained Q111 [34]. Together with the occurrence of Q111E in the black-headed grosbeak (*Pheucticus melanocephalus*) [26], the present finding indicates that Q111E occurs in multiple, distantly related avian lineages.

Comparison of the crested serpent-eagle and grey-faced buzzard further provides insight into the sequence backgrounds in which Q111E occurs. Although both species possess Q111E/N122, they differ at several residues surrounding position 111, including positions 108, 114, 115, 116, 117, 118, and 120. In particular, several M1–M2 amino acid states previously observed in the crested serpent-eagle and black-chested snake-eagle were absent from the grey-faced buzzard. The specific M1–M2 sequence background of the crested serpent-eagle therefore does not appear to be required for the occurrence of Q111E. This observation is relevant because experimental studies in insects and amphibians have demonstrated that the phenotypic effects and functional costs of resistance-associated substitutions can depend on the surrounding NKAα sequence [27,52,53]. Our previous study also detected evidence of selection at residues other than position 111 in the crested serpent-eagle [34]. Broader taxonomic sampling, particularly among close relatives of the crested serpent-eagle and grey-faced buzzard, together with ancestral-state reconstruction and functional analyses, will be necessary to determine whether Q111E evolved independently in these lineages and to assess how its effects may depend on the surrounding sequence background.

Until such evidence is available, Q111E should be regarded as a candidate molecular signature of potential target-site insensitivity rather than as evidence of CTS resistance.

#### 3.3.3. Conservation Implications of Molecular Screening for Cane Toad Vulnerability

Inferring resistance to cane toad toxins from NKAα1 sequence information alone has important limitations. Target-site insensitivity represents only one component of resistance, and the effects of individual substitutions may depend on the surrounding amino acid background [27,52,53]. In addition, an NKAα1-only screen does not capture resistance mediated by other paralogs, particularly NKAα3-associated substitutions reported in squamate reptiles [28,29,57,67]. Candidate substitutions in NKAα1 should therefore not be interpreted as evidence of organismal CTS resistance. An additional limitation is that much of the experimental evidence for resistance-associated substitutions is based on assays using ouabain or individual CTS compounds, whereas cane toad secretions contain a chemically diverse mixture of bufadienolides [15]. Resistance to a particular CTS therefore cannot necessarily be extrapolated to resistance to cane toad toxins as a whole. Whole-animal susceptibility can also be influenced by toxin absorption, distribution, metabolism, and excretion, as well as behavior, prey-handling strategies, and other physiological mechanisms [31,51,57,68,69,70]. Accordingly, the absence of a recognized resistance-associated NKAα1 state cannot be taken as direct evidence of susceptibility, just as the presence of a candidate substitution does not demonstrate that an animal can consume cane toads without adverse effects.

Nevertheless, this review provides an initial framework for identifying species and knowledge gaps that warrant further investigation under current or potential exposure to invasive cane toad toxins. The predominance of the ancestral Q111/N122 state among the endangered species examined here does not itself indicate susceptibility, but emphasizes how little can currently be inferred about their physiological responses from available molecular information alone.

This issue is particularly important for the Iriomote cat (MOEJ: CR). Its frequent consumption of anurans and the high biomass of amphibians on Iriomote-jima Island indicate a potentially high probability of dietary exposure should cane toads become established [71,72,73,74,75]. The Iriomote cat therefore represents a priority species for further investigation. Its Q111/N122 state provides no evidence of recognized target-site insensitivity, but neither does it demonstrate physiological susceptibility. Functional assays using Iriomote cat NKAα1, followed by physiological and toxicological studies where ethically feasible, would be required to determine whether the molecular state observed here translates into sensitivity to cane toad toxins. Cane toads have not yet become established on Iriomote-jima Island, but previous incursions have been recorded [17,23]. Preventing their establishment remains a conservation priority based on the known ecological impacts of cane toads and existing precautionary management objectives [12,16,17,22,76,77], particularly given the risk of accidental introduction through the frequent movement of people and goods by ship from Ishigaki-jima Island. This priority is independent of the genetic screening conducted here, which should instead be viewed as a means of identifying species and knowledge gaps that warrant further investigation should exposure occur.

The same caution applies to Ishigaki-jima Island, where cane toads are already established and some native predators are known to exploit them successfully. In addition to the crested serpent-eagle, several bird species, including the Malayan night heron (*Gorsachius melanolophus*), have occasionally been observed capturing cane toads (M. Kobayashi, personal communication; Figure S1). These observations provide opportunities to investigate the molecular, physiological, and behavioral mechanisms that enable native predators to exploit toxic prey. The large-billed crow provides a particularly informative example because repeated cane toad consumption has been observed despite the absence of currently recognized resistance-associated substitutions at positions 111 and 122 in its NKAα1 sequence.

On Ishigaki-jima Island, we have frequently observed crows feeding on road-killed cane toads. Individuals have also been observed apparently avoiding the parotoid region while feeding (A.T., personal observation; Figure S2), suggesting that behavioral avoidance may reduce toxin exposure, similar to toxin-avoidance behavior previously reported in Torresian crows (*Corvus orru*) [78]. However, avoidance of the parotoid glands does not necessarily eliminate toxin exposure because toxin-secreting glands also occur on the limbs and dorsal skin [79]. Repeated consumption may therefore expose crows to lower doses of CTSs even when the most toxin-rich tissues are avoided [80]. Their ability to exploit cane toads may consequently involve behavioral avoidance, physiological tolerance, or a combination of mechanisms that cannot be inferred from NKAα1 sequence variation alone. Whether repeated exposure also causes sublethal or cumulative effects remains unknown and should be investigated through measurements of CTS exposure, tissue burdens, and physiological responses.

Furthermore, the two captive species examined here, the domestic dog (*Canis lupus familiaris*) and domestic cat (*Felis catus*), retained the Q111/N122 state. Together with previous reports of clinical poisoning caused by cane toads in dogs and cats in Australia [81,82,83], this finding highlights the need to consider the risk of cane toad exposure to companion animals on Ishigaki-jima Island. The documented susceptibility of domestic cats is also relevant when considering potential risks to the Iriomote cat, a closely related felid. Although susceptibility cannot be inferred directly from domestic cats to the Iriomote cat, documented cane toad poisoning in another felid provides a relevant comparative basis for prioritizing direct investigation of the Iriomote cat’s physiological response to cane toad toxins.

More broadly, our screening highlights substantial gaps in the genetic information required even for this initial stage of vulnerability assessment. NKAα1 sequences were available for only approximately half of the native vertebrate species considered, with particularly limited representation of reptiles and amphibians. This uneven coverage also reflects broader taxonomic disparities in the availability of genomic resources, highlighting the need to expand reference genomic data across underrepresented lineages [84]. Species prioritized for further investigation through such screening require subsequent functional, toxicological, ecological, and ultimately demographic evaluation to determine whether molecular differences translate into population-level vulnerability. Expanding baseline genomic and ecological information for island fauna will be important not only for assessing potential impacts of cane toads but also for anticipating responses to future biological invasions and other emerging threats.

## 4. Conclusions

The ecological effects of cane toad toxins should not be evaluated solely in terms of conspicuous acute mortality. Species may differ in prey-handling behavior, exposure dose, physiological detoxification, target-site sensitivity, and responses to repeated sublethal exposure, potentially resulting in outcomes ranging from avoidance and successful exploitation to chronic physiological effects or lethal poisoning. Molecular screening can identify amino acid states associated with potential target-site insensitivity and thereby help identify questions for further investigation, but cannot by itself predict whole-animal susceptibility or conservation risk. Continued monitoring of predator–toad interactions, together with functional and toxicological studies, will therefore be important on Ishigaki-jima Island. For Iriomote-jima Island, where cane toads have not become established, preventing their establishment remains the primary conservation priority. The predominance of NKAα1 states without currently recognized resistance-associated substitutions among the species examined here highlights substantial uncertainty regarding their physiological responses to cane toad toxins. Resolving this uncertainty will require integrating molecular evidence with functional, toxicological, ecological, and ultimately demographic information.

## Figures and Tables

**Table 1:** Representative experimental studies identifying amino acid substitutions that affect CTS sensitivity of NKA.

Substitution (s)	Experimental System	CTS Tested	Reported Effects on CTS Sensitivity	Ref.
Q111R/N122D	Sheep NKAα1 carrying the indicated mutation, expressed in HeLa cells	Ouabain	~100-fold increase in resistance (NKA activity)	[30]
Q111D/N122R	Sheep NKAα1 carrying the indicated mutation, expressed in HeLa cells	Ouabain	5 × 10^4^-fold increase in resistance (NKA activity)	[36]
Q111K/N122K			5 × 10^3^-fold increase in resistance (NKA activity)	
Q111R/N122D			1 × 10^3^-fold increase in resistance (NKA activity)	
Q111E/N122E			100-fold increase in resistance (NKA activity)	
Q111K/N122E			60-fold increase in resistance (NKA activity)	
N122D			10-fold increase in resistance (NKA activity)	
Q111R			10-fold increase in resistance (NKA activity)	
C104A	Sheep NKAα1 carrying the indicated mutation, expressed in HeLa cells	Ouabain	6-fold increase in resistance (NKA activity)	[37]
C104F			150-fold increase in resistance (NKA activity)	
Y108A			9-fold increase in resistance (NKA activity)	
R880P	Sheep NKAα1 carrying the indicated mutation, expressed in HeLa cells	Ouabain	5.2-fold increase in *K_D_* (reduced ouabain-binding affinity)	[38]
C104A			2.9-fold increase in *K_D_* (reduced ouabain-binding affinity)	
Y108A			4.3-fold increase in *K_D_* (reduced ouabain-binding affinity)	
P118K			4.0-fold increase in *K_D_* (reduced ouabain-binding affinity)	
T797I	Mouse NKAα1 carrying a point mutation expressed in African green monkey kidney (CV-1) cells	Ouabain	63-fold increase in resistance (Rb^+^ uptake)	[39]
T797A	Sheep NKAα1 carrying the indicated mutation, expressed in HeLa cells	Ouabain Ouabagenin Dihydroouabain Digoxin Digitoxin	67-fold increase in resistance to ouabain (NKA activity)	[40]
T797V			79-fold increase in resistance to ouabain (NKA activity)	
L793P	Sheep NKAα1 carrying the indicated mutation, expressed in HeLa cells	Ouabain	24-fold increase in resistance (NKA activity)	[41]
L793N			2.8-fold increase in resistance (NKA activity)	
L793K			8.8-fold increase in resistance (NKA activity)	
F786I			11-fold increase in resistance (NKA activity)	
F786N			19-fold increase in resistance (NKA activity)	
F863L			5.7-fold increase in resistance (NKA activity)	
L330Q	Sheep NKAα1 carrying the indicated mutation, expressed in HeLa cells	Ouabain	7.9-fold increase in resistance (NKA activity)	[42]
A331G			3.1-fold increase in resistance (NKA activity)	
T334P/A331G			6.5-fold increase in resistance (NKA activity)	
T338N			3.3-fold increase in resistance (NKA activity)	
T338A			3.2-fold increase in resistance (NKA activity)	
F982S			6.3-fold increase in resistance (NKA activity)	

**Table 2:** Functional effects of naturally evolved NKAα substitutions associated with CTS resistance.

Substitution (s)	NKAα Paralog	Evidence/Experimental System	CTS Tested	Reported Association or Functional Effect	Ref.
Q111L	NKAα	Comparative survey of 18 adapted insect species; *Drosophila melanogaster* NKAα variants carrying the indicated substitutions expressed in HeLa cells.	Ouabain	No significant increase in cell survival relative to WT at 0.5 μM ouabain	[49]
Q111T				No significant increase in cell survival relative to WT at 0.5 μM ouabain	
Q111V				Significantly increased cell survival relative to WT at 0.5 μM ouabain	
N122H				Significantly increased cell survival relative to WT at 0.5 μM ouabain	
Q111T/N122H				>12-fold increase over WT and >2-fold greater survival rate than either substitution alone at 0.5 μM ouabain	
L111V	NKAα	Native NKA from Danaini butterflies differing naturally at sites 111/122	Ouabain	Mean IC_50_ increase from 1.47 × 10^−6^ M (L111/N122) to 4.96 × 10^−6^ M (V111/N122): ~3.4-fold increase in resistance (NKA activity)	[51]
L111V/N122H		Native NKA from *Danaus plexippus*		IC_50_ = 9.87 × 10^−5^ M versus 1.47 × 10^−6^ M for L111/N122 Danaini: ~67-fold increase in resistance (NKA activity); ~20-fold relative to V111/N122	
N122H	NKAα	*D. melanogaster* NKAα with each mutation expressed in Sf9 cells	Ouabain	IC_50_ 61.0 μM versus 0.24 μM WT: ~254-fold increase in resistance (NKA activity)	[54]
T797A				IC_50_ 63.3 μM versus 0.24 μM WT: ~264-fold increase in resistance (NKA activity)	
Q111V/N122H				IC_50_ 550 μM versus 0.24 μM WT: ~2290-fold increase in resistance (NKA activity)	
Q111T/N122H				IC_50_ 583 μM versus 0.24 μM WT: ~2430-fold increase in resistance (NKA activity)	
Q111L/A119S	NKAα	Endogenous *D. melanogaster*, genome-edited	Ouabain	7-fold increase in NKA insensitivity relative to WT; A119S partially ameliorated neurological dysfunction associated with site-111 substitutions	[52]
Q111V/A119S				11-fold increase in NKA insensitivity relative to WT; A119S ameliorated Q111V-associated neurological dysfunction	
Q111T/A119S				28-fold increase in NKA insensitivity relative to WT	
A119S				1.45-fold increase in NKA insensitivity, accompanied by a substantial increase in adult survival during ouabain exposure	
A119S/N122H				178-fold increase in NKA insensitivity relative to WT; A119S partially ameliorated N122H-associated neurological dysfunction	
Q111L/A119S/G120R	NKAα1	Varanid H1–H2 sequence expressed in HEK293 cells	Bufalin	Resistant Asian–African varanid sequence showed ~3000-fold greater resistance than susceptible Australian varanid sequence	[28]
Q111R	NKAα1	Susceptible *Leptodactylus* NKAα1 engineered with resistance-associated substitutions; expressed in Sf9 cells	Ouabain	~5.5-fold increase in resistance; ATPase activity reduced by ~40%	[27]
N122D				~3.7-fold increase in resistance; ATPase activity reduced by ~40%	
Q111R/N122D				~102-fold increase in resistance; ATPase activity reduced by ~30%	
A112T/E116D/I135V/ L180Q/I199L/I279V/ S403C/L536M/Q701L/I788M				No significant increase in resistance (*p* = 0.22); ATPase activity essentially unchanged relative to susceptible NKAα1	
Q111R/N122D + above 10 substitutions				~251-fold increase in resistance; additional substitutions substantially ameliorated the activity cost associated with Q111R/N122D	

## Data Availability

The sequence data analyzed in this study are publicly available from the National Center for Biotechnology Information (NCBI); accession information and the NKAα1 protein sequences used for comparative analyses are provided in the Supplementary Materials.

## Supplementary Materials

The following supporting information can be downloaded at: https://www.mdpi.com/article/10.3390/genes17101286/s1, Table S1. Terrestrial vertebrate species occurring on Ishigaki-jima and Iriomote-jima Islands included in the survey of NKAα1 sequences; Table S2. Amino acid states at 41 NKAα1 positions previously implicated in cardiotonic steroid sensitivity among vertebrates examined in this study; Table S3. Comparison of experimentally implicated NKAα1 residues with amino acid variation observed among vertebrates examined in this study; Table S4. Amino acid states at NKAα1 positions examined in functional studies summarized in Table 2; Table S5. Comparison of amino acid variation in vertebrate NKAα1 with substitutions examined in the functional studies summarized in Table 2; Figure S1. Predation on a cane toad by a Malayan Night Heron (*Gorsachius melanolophus*) on Ishigaki-jima Island, Japan; Figure S2. Predation on a road-killed cane toad by a Large-billed Crow (*Corvus macrorhynchos osai*) on Ishigaki-jima Island, Japan; Supplementary File S1. Multiple protein alignment of NKAα1 and reference NKAα paralogs used for phylogenetic and comparative analyses; Supplementary File S2. Maximum-likelihood phylogenetic tree of NKAα sequences used to confirm NKAα1 orthology.

## Abbreviations

The following abbreviations are used in this manuscript:

CTSs	Cardiotonic steroids
BDs	Bufadienolides
NKA	Na+/K+-ATPase
NCBI	National Center for Biotechnology Information
