# Supplementary material for: Molecular Screening for Potential Resistance to Toxic Invasive Prey: Implications for Island Conservation
Source: Genes (Basel). 2026 Oct 15;17(10):1286. doi: 10.3390/genes17101286 (PMC13704064; doi:10.3390/genes17101286)
Supplement: Supplementary file 1 [file genes-17-01286-s001.zip › Supplementary file/Supplementary_Figures.pdf]

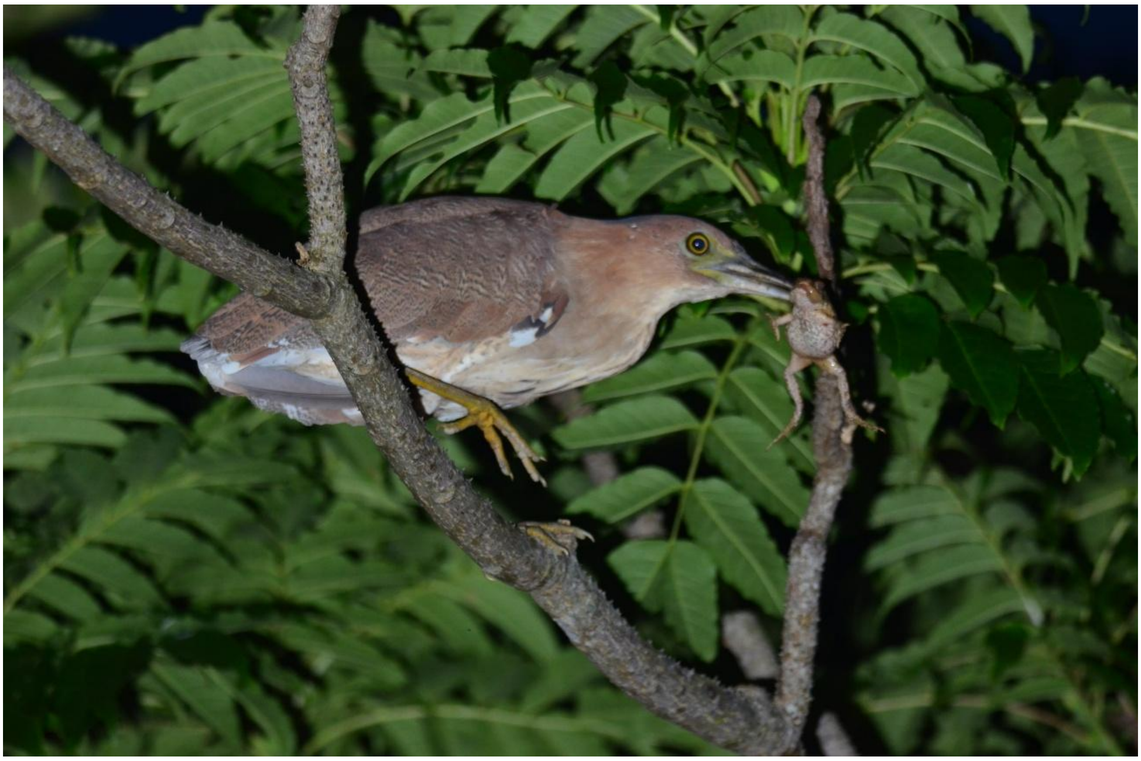

Figure S1. Predation on a cane toad by a Malayan Night Heron (*Gorsachius melanolophus*) on Ishigaki-jima Island, Japan. The photograph was taken by Masahiro Kobayashi on 16 June 2016.

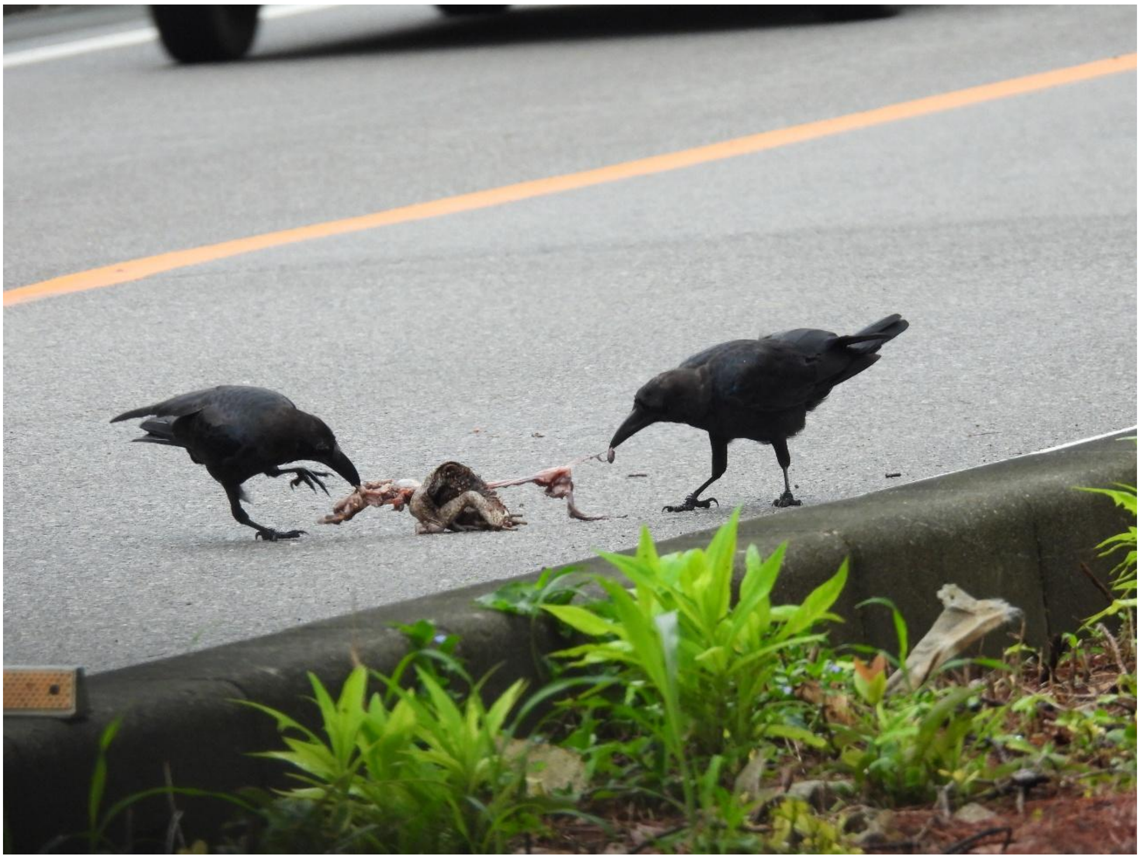

Figure S2. Predation on a road-killed cane toad by a Large-billed Crow (*Corvus macrorhynchos osai*) on Ishigaki-jima Island, Japan. The photograph was taken by Alisa Tobe on 4 September 2026.
